# The Involvement of Macrophage Colony Stimulating Factor on Protein Hydrolysate Injection Mediated Hematopoietic Function Improvement

**DOI:** 10.3390/cells10102776

**Published:** 2021-10-16

**Authors:** Shimiao Wang, Yuchong Zhang, Weiqi Meng, Yihao Dong, Sujie Zhang, Lesheng Teng, Yang Liu, Lanzhou Li, Di Wang

**Affiliations:** 1 School of Life Sciences, Jilin University, Changchun 130012, China; wangsm19@mails.jlu.edu.cn (S.W.); mengwq19@mails.jlu.edu.cn (W.M.); tenglesheng@jlu.edu.cn (L.T.); 2 Engineering Research Center of Chinese Ministry of Education for Edible and Medicinal Fungi, College of Plant Protection, Jilin Agricultural University, Changchun 130118, China; zhangyuchong163@163.com (Y.Z.); dyh1442811205@163.com (Y.D.); zhangsujiejlau@163.com (S.Z.); y_liu10@jlau.edu.cn (Y.L.)

**Keywords:** protein hydrolysate injection, hematopoietic function, M-CSF

## Abstract

Protein hydrolysate injection (PH) is a sterile solution of hydrolyzed protein and sorbitol that contains 17 amino acids and has a molecular mass of 185.0–622.0 g/mol. This study investigated the effect of PH on hematopoietic function in K562 cells and mice with cyclophosphamide (CTX)-induced hematopoietic dysfunction. In these myelosuppressed mice, PH increased the number of hematopoietic cells in the bone marrow (BM) and regulated the concentration of several factors related to hematopoietic function. PH restored peripheral blood cell concentrations and increased the numbers of hematopoietic stem cells and progenitor cells (HSPCs), B lymphocytes, macrophages, and granulocytes in the BM of CTX-treated mice. Moreover, PH regulated the concentrations of macrophage colony stimulating factor (M-CSF), interleukin (IL)-2, and other hematopoiesis-related cytokines in the serum, spleen, femoral condyle, and sternum. In K562 cells, the PH-induced upregulation of hematopoiesis-related proteins was inhibited by transfection with M-CSF siRNA. Therefore, PH might benefit the BM hematopoietic system via the regulation of M-CSF expression, suggesting a potential role for PH in the treatment of hematopoietic dysfunction caused by cancer therapy.

## 1. Introduction

Myelosuppression is a significant side effect of most chemotherapeutic and radiotherapeutic agents [1], leading to decreased red and white blood cell populations [2] and potential immunosuppression [3]. Fatal infections may occur in severe cases [4]. Thus, myelosuppression significantly affects patient outcomes. Hematopoiesis refers to the highly coordinated process of blood cell development and homeostasis [5]. HSPCs, located in the bone marrow, fail to proliferate sufficiently in myelosuppressed patients [6]. Hematopoiesis involves the self-renewal of hematopoietic stem cells (HSCs), proliferation of hematopoietic progenitor cells (HPCs), and maturation of differentiated cells [7]. Therefore, myelosuppression leads to significant hematopoietic dysfunction [6].

Cyclophosphamide (CTX), one of the most commonly used alkylating cytotoxic chemotherapeutics, is often used to treat various types of autoimmune diseases and is used in combination with chemotherapy in cancer treatment [8]. CTX treatment can lead to myelosuppression due to its lack of cell-type specificity, negatively affecting hematopoietic precursor stem cells [9]. As a result, CTX is often used to induce hematopoietic dysfunction in vivo models [10]. The main symptoms of CTX-induced hematopoietic dysfunction are weight loss, decreased numbers of peripheral blood cells, and reduced white blood cell (WBC) counts [9]. Our previous study found that CTX reduces the circulating levels of macrophage-colony stimulating factor (M-CSF) and interleukin (IL)-2 [11]. M-CSF stimulates the formation of macrophage colonies from bone marrow HPCs. IL-2, produced by activated T lymphocytes, not only promotes the growth and differentiation of B lymphocytes cells [12], but can also improve hematopoietic function [13]. Accordingly, IL-2 may enhance the production of M-CSF directly or indirectly [14].

Currently, recombinant human granulocyte colony-stimulating factor (rhG-CSF), granulocyte colony-stimulating factor (G-CSF), and erythropoietin (EPO) are used to treat therapy-associated hematopoietic dysfunction. Specifically, rhG-CSF is used to treat hematopoietic dysfunction in cancer patients who have received chemotherapy and radiotherapy [15]. However, such therapies are limited by high costs and significant side effects. Treatment with myeloid growth factors can lead to severe bone pain, while treatment with EPO-stimulating agents can increase the risks of stroke, myocardial infarction, and cardiac failure [16]. Thus, a safe and broadly applicable treatment strategy is urgently needed for the clinical management of hematopoietic dysfunction. In our previous studies, we reported improved hematopoietic function following treatment with polypeptide biologics, such as calf spleen extractive injection (State Food and Drug Administration (SFDA) Approved NO.: H22026121) [17] and calf thymus polypeptide (SFDA Approved NO.: H20065575) [11]. Several protein hydrolysates have also been reported to exert anti-hypertensive, anti-cancer, anti-bacterial, and insulinogenic effects [18,19]. Amino acids have been reported to not only play a role as nutrients, but also possess unique biological activities. PH (SFDA Approved NO.: H22026405) is a sterile solution of hydrolysate casein and lactalbumin. Clinically, PH is predominantly used for the treatment of severe surgical trauma, severe amino acid deficiency caused by large burns, and hypoproteinemia caused by various diseases. Although PH has been clinically used for years, only its anti-oxidative pharmacological properties have been examined [20,21]. To date, there has been no systematic study of the molecular basis of PH, and it remains unclear exactly how PH exerts its beneficial effects on a molecular level.

Based on the clinical usage of PH, we hypothesized that it may be beneficial in the treatment of hematopoietic dysfunction caused by chemotherapy or radiotherapy. In this study, we examined the effects of PH on hematopoietic function in K562 cells and mice with CTX-induced hematopoietic dysfunction. We found that PH improved hematopoietic function, potentially via the regulation of M-CSF and other hematopoietic related factors.

## 2. Materials and Methods

### 2.1. PH Preparation

PH (SFDA Approved NO.: H22026405) is a sterile hydrolyzed casein hydrolysate and lactalbumin hydrolysate suitable for intravenous injection. It was provided by Liaoyuan Hongyuan Pharmaceutical Co., Ltd. (Liaoyuan, China). The same batch of PH (Batch number: 20071801) was applied in the whole experiment.

### 2.2. Molecular Mass and Amino Acid Analyses

The Viscotek TDA 305 multidetector gel permeation chromatography system (Malvern Panalytical Ltd., Malvern, UK) was used to determine the molecular weight distribution of PH, using an SRT SEC-300 gel filtration column (30 cm × 4.6 mm, 5 μm). The column temperature was maintained at 30 °C, and the injection volume of the sample was 100 μL. The mobile phase was 0.1 M pH 7.0 phosphate-buffered saline (PBS), and the elution rate was 0.5 mL/min. The amino acid content of PH was determined using a high-performance liquid chromatograph (HPLC) (Agilent 1100, Agilent Technologies, Inc., Böblingen, Germany) equipped with a Diamonsil C18 (250 mm × 4.6 mm, 5 μm) chromatographic column [22]. The detection wavelength was 360 nm, mobile phase A was 0.05 mol/L of sodium acetate solution (containing 1% N, N-dimethyl formaldehyde; pH 6.4) and mobile phase B was acetonitrile-water (1:1). The column temperature was maintained at 40 °C, and the sample injection volume was 20 μL with a flow rate of 1.0 mL/min. The amino acid contents are expressed in grams of each amino acid per 100 g of protein.

### 2.3. Cell Experiments

#### 2.3.1. Cell Culture

Human myelogenous leukemia K562 (CCL-243^™^) cells were purchased from the American Type Culture Collection (ATCC, Manassas, VA, USA). The cells were cultured in Roswell Park Memorial Institute (RPMI) medium 1640 (Gibco, Invitrogen, Carlsbad, CA, USA) supplemented with 10% fetal bovine serum (FBS, Procell Life Science & Technology Co., Ltd., Wuhan, China), 100 U/mL penicillin and 100 μg/mL streptomycin (Gibco) at 37 °C in a humidified incubator with 5% CO_2_ and 95% air.

#### 2.3.2. Transfection of M-CSF siRNA in K562 Cells

For the siRNA-mediated knockdown, the K562 cells were transfected with M-CSF siRNA using LipoRNAi™ Transfection Reagent (Beyotime Technology, Shanghai, China). The K562 cells were seeded in a six-well plate at a density of 2.5 × 10^5^ cells/well, and transfection was carried out at a confluence level of 70–80%. The cells were co-transfected with 121 μL of 1640 basic medium, 100 pmol of M-CSF siRNA (GGATGAAGAGACCGGAGAA) (siG11517142729, RiboBio, Guangzhou, China) and 4 μL of LipoRNAi^™^ Transfection Reagent. Then, the cells were incubated with 0 or 20 mg/mL PH for 24 h, as per the manufacturer’s guidelines, and the expression levels of M-CSF, p90 ribosomal S6 kinases 1 (RSK1p90), phosphorylated (P)-RSK1p90, c-Myc, extracellular regulated protein kinases 1/2 (ERK1/2), and P-ERK1/2 (Table 1) were assessed using Western blots.

### 2.4. Animal Experiments and Drug Treatment Protocols

Male BALB/c mice (4–6 weeks old, 20 ± 2 g, specific pathogen-free (SPF) grade; SCXK (Liao) 2020-0001) were purchased from Liaoning Changsheng Biotechnology Co., Ltd. (Liaoning, China). The mice were housed in a strictly controlled environment, with a 12/12 h light/dark cycle (light on from 08:00 to 20:00), temperature of 23 ± 1 °C and relative humidity of 50% ± 10%. Sterile food and water were provided ad libitum. The experimental protocol was approved by the Institutional Animal Care and Use Committee of Jilin University (SY202009001).

After a 7-day adaptation period, 60 mice were intraperitoneally injected with CTX (100 mg/kg, Sigma Aldrich, St. Louis, MO, USA) dissolved in normal saline (NS) once daily for 3 days to induce hematopoietic dysfunction. The mice were randomly divided into four groups and intraperitoneally injected with either 0.2 mL/20 g of NS (model group; *n* = 15); 3.25 (*n* = 15) or 6.5 g/kg of PH (*n* = 15) once daily for 4 weeks; or 30 μg/kg of rhG-CSF (*n* = 15) (Qilu Pharmaceutical Co., Ltd., Jinan, China) twice weekly for 4 weeks. The mice were also injected with CTX (80 mg/kg) once weekly to avoid the recovery of hematopoietic function. The remaining 30 mice were intraperitoneally injected with NS for 3 days, after which they were injected once daily with 0.2 mL/20 g of NS (control group; *n* = 15) or 6.5 g/kg of PH (PH treatment group; *n* = 15) for 4 weeks.

On the 1st, 4th, 11th, 18th, 25th, and 32nd days, mouse bodyweights were measured and recorded (Figure S1). After sample collection, the mice were euthanized by CO_2_ inhalation (LY-FL-1, Lingyunboji Technology, Beijing, China), and the liver, spleen, kidney, and thymus were collected and weighed. Organ indexes were calculated as follows: organ index (%) = organ weight (mg)/bodyweight (g). The femur and tibia were removed in a sterile environment.

### 2.5. Assessment of Peripheral Blood Physiological Indexes

Two hours after the final drug administration, blood samples were taken from the caudal vein. Changes in the composition of the peripheral blood samples were immediately assessed using an automatic blood analyzer (Drew Scientific Group, Dallas, TX, USA). Blood samples were randomly collected from five mice in each group, and the analysis was repeated three times.

### 2.6. Flow Cytometry

Flow cytometry (CytoFLEX 488 nm) was used to determine the proportions of HSPCs, B lymphocytes, macrophages, and granulocytes in bone marrow-derived mononuclear cells (BMMNCs) samples of mice with hematopoietic dysfunction. Sca-1, a member of the Ly-6 multi-gene family also known as Ly-6A/E, is a glycosylphosphatidylinositol (GPI) connexin expressed on HSPCs. The expression levels of the established HPSCs markers Sca-1 and c-Kit on Lin^−^ cells (Lin^−^/Sca-1^+^/c-Kit^+^), were quantified to examine the effects of PH on the differentiation potential of bone marrow cells. CD45 and CD19 are specific surface markers used to identify B lymphocytes (CD45^+^CD19^+^) [23] in BMMNCs samples. In mice, granulocytes and monocytes typically express Gr1 and CD11b, while monocytes express high levels of F4/80 and granulocytes express Ly6G [24]. The proportions of macrophages (CD11b^+^F4/80^+^) and granulocytes (CD11b^+^Ly6G^+^) in mouse BMMNCs samples were thus evaluated according to the expression levels of CD11b, F4/80 and Ly6G. Five mice per group were randomly selected, and BMMNCs were detected. The protocols followed were the same as in our previous studies [25]. Antibodies are shown in Table 1.

### 2.7. Histopathological Analysis

Tissues, including heart, liver, spleen, and kidney, were collected randomly from three mice in each group and fixed in 4% paraformaldehyde for 48 h. The sternum and femoral condyles were collected and fixed in 4% paraformaldehyde for 48 h and decalcified in 10% ethylenediaminetetraacetic acid for 7 days. All samples were embedded in paraffin with 10% neutral buffered formalin, washed with water, dehydrated in ascending grades of alcohol, clarified with xylene, and finally embedded in paraffin. After slicing into 5-μm sections, all samples were stained in hematoxylin and eosin (H&E). The sternum and femoral condyle samples were also stained with antibodies specific for M-CSF and IL-2 (Table 1). All samples were examined under a light microscope (BX51; Olympus, Tokyo, Japan). ImageJ software (National Institutes of Health, Bethesda, MD) was used to quantify the pixel density for semi-quantitative densitometric analysis of protein expressions.

### 2.8. Cytokine Assays

Commercial enzyme-linked immunosorbent assay (ELISA) kits (Boster Biological Technology Co., Ltd., Wuhan, China) were used to determine the concentrations of macrophage inflammatory protein (MIP)-1α (EK0449), tumor necrosis factor (TNF)-α (EK0527), and M-CSF (EK0445) in the serum of mice in each group. ELISAs were carried out according to the manufacturer’s protocols. Blood samples were collected from the caudal veins of six randomly chosen mice in each group, and quantifications were repeated three times.

### 2.9. Western Blotting

Based on the histopathological analyses, Western blots were used to determine the expression levels of M-CSF and IL-2 in spleen tissue collected from six randomly selected mice in each group. Proteins were extracted from K562 cells (five samples) and spleen tissue using radioimmunoprecipitation assay lysis buffer supplemented with 1% protease inhibitor cocktail (Sigma-Aldrich, St. Louis, MO, USA) and 2% phenylmethanesulfonylfluoride (Sigma-Aldrich). Total protein concentrations were quantified using a bicinchoninic acid (BCA) assay kit (Thermo Fisher Scientific, Shanghai, China). Proteins (40 μg per group) were separated by 10% or 12% sodium dodecyl sulfate polyacrylamide gel electrophoresis (SDS-PAGE) and transferred to polyvinylidene difluoride (PVDF) membranes (0.45 μm, GE Healthcare, Pittsburgh, USA). The membranes were blocked at room temperature with NcmBlot Blocking Buffer (NCM Biotech, Suzhou, China) for 30 min and incubated with primary antibodies (Table 1) at 4 °C overnight. After washing the membranes with triethanolamine-buffered saline (TBS) containing Tween 20 (TBST), the membranes were incubated with horseradish peroxidase (HRP)-conjugated secondary antibodies (Table 1) at 4 °C for four hours. An enhanced chemiluminescence detection kit (GLPBIO, Montclair, NJ, USA) and imaging system (Tanon-5200, Tanon Science & Technology Co., Ltd., Shanghai, China) were used to visualize the resultant protein bands. ImageJ software (National Institutes of Health) was used to quantify the pixel density for semi-quantitative densitometric analysis of protein concentrations. Quantification data were normalized by GAPDH, and reported as the folds of those from the corresponding control mice.

### 2.10. Statistical Analysis

Statistical significance was determined using a one-way analysis of variance (ANOVA) followed by a Tukey’s post-hoc test using BONC DSS Statistics 25 software (Business-intelligence of Oriental Nations Co., Ltd., Beijing, China). Data are shown as the mean ± standard deviation (SD), and results were considered significant at a *p* value < 0.05.

## 3. Results

### 3.1. Molecular Mass Distribution and Amino Acid Content of PH

The specific molecular components of PH may be responsible for its unique pharmacological activities; however, to date, there have been no systematic studies of the composition of PH. Therefore, the molecular mass distribution and amino acid content of PH were initially determined. Accordingly, 2.5% of PH had a molecular mass of 475.0–622.0 g/mol, while 97.5% of PH had a molecular mass <475.0 g/mol (Table 2). Seventeen different amino acids were detected, with the concentrations of glutamic acid (0.64 g/100 g), leucine (0.42 g/100 g), lysine (0.45 g/100 g), and proline (0.41 g/100 g) being much higher than those of other amino acids (Table 3). Specific peaks are illustrated in Figure S2.

### 3.2. PH promoted the Hematopoietic Function of Mice

**Figure 1:**
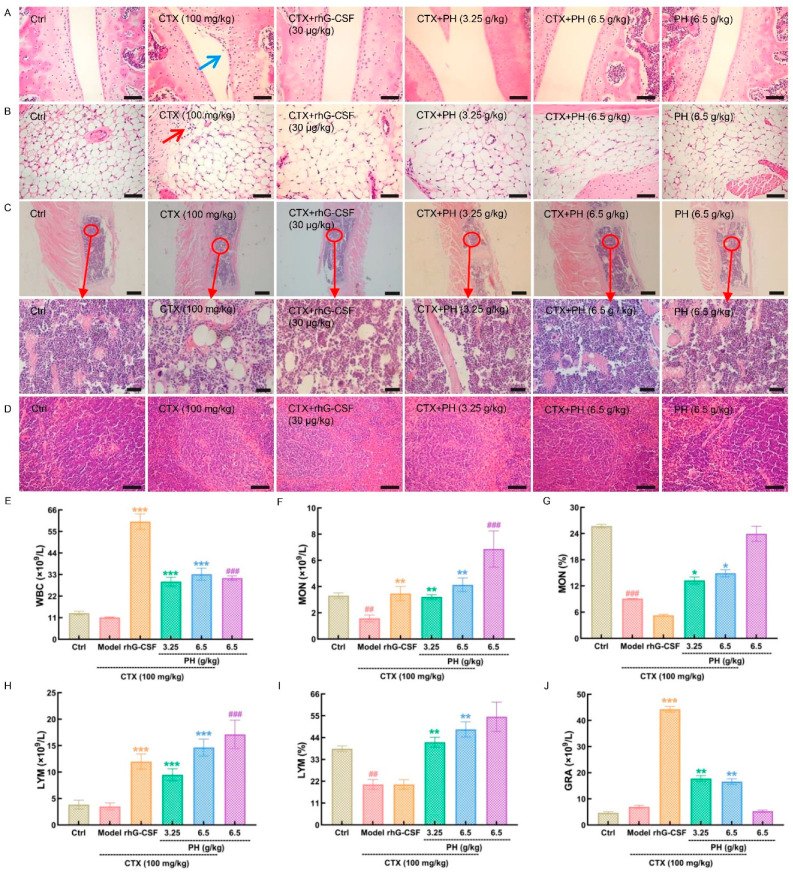
The protection effect of PH on CTX-induced mice with hematopoietic dysfunction. H&E staining was used to evaluate pathological alterations of (**A**) femoral condyle joint cavity (200×, scale bar: 100 μm), (**B**) joint circumference (200×, scale bar: 100 μm), (**C**) sternal marrow cavity (40×, scale bar: 400 μm; 400×, scale bar: 40 μm), and (**D**) spleen (200×, scale bar: 100 μm) under a light-microscope digital camera (*n* = 3 mice/group in triplicate). Blue arrow in (**A**), a depression with inflammatory cell infiltration on the joint surface. Red arrow in (**B**), inflammatory cell infiltration can be seen in the tissue around the joint. PH regulated the levels of (**E**) WBC, (**F**,**G**) MON, (**H**,**I**) LYM, and (**J**) GRA in peripheral blood. Data are showed as the mean ± SD (*n* = 5 mice/group in triplicate) and determined via a one-way ANOVA followed by a Tukey’s post hoc test comparison. ^##^ *p* < 0.01 and ^###^ *p* < 0.001 vs. control group, * *p* < 0.05, ** *p* < 0.01 and *** *p* < 0.001 vs. model group. CTX, cyclophosphamide; Ctrl, control; Model, hematopoietic dysfunction model; rhG-CSF, recombinant human granulocyte colony-stimulating factor; PH, Protein hydrolysate injection; WBC, white blood cell; LYM, lymphocytes; MON, monocytes; and GRA, granulocytes.

In mice, general health and hematopoietic function can be determined according to body weight and organ indexes. Four weeks of treatment with PH and rhG-CSF increased the body weights of mice with hematopoietic dysfunction (*p* < 0.01). It also alleviated the pathological changes in organ indexes (*p* < 0.05), especially in the spleen (*p* < 0.001) and thymus (*p* < 0.05). PH alone had no significant effect on body weight or organ indexes in healthy mice (Table S1). According to H&E staining, there was a depression with inflammatory cell infiltration on the joint surface of CTX-induced mice with hematopoietic dysfunction (Figure 1A, blue arrow). Moreover, the inflammatory cell infiltration can also be seen in the tissue around the joint of CTX-induced mice with hematopoietic dysfunction (Figure 1B, red arrow). rhG-CSF and PH significantly reduced inflammatory cell infiltration in the femoral condyle joint cavity (Figure 1A) and joint circumference (Figure 1B) and the concentration of cells and proportion of vacuoles in the sternal marrow cavity (Figure 1C), and restored the structure of splenic cells (Figure 1D) in mice with CTX-induced hematopoietic dysfunction. There were no obvious pathological changes in the heart, liver, or kidney in any experimental group (Figure S3).

Bone marrow dysfunction can be evaluated using physiological indexes of peripheral blood [26,27]. In mice with hematopoietic dysfunction, PH significantly enhanced the number of WBCs (*p* < 0.001) (Figure 1E), number of monocytes (MONs) (*p* < 0.01) (Figure 1F), percentage of MONs (*p* < 0.05) (Figure 1G), number of lymphocytes (LYMs) (*p* < 0.001) (Figure 1H), percentage of LYMs (*p* < 0.01) (Figure 1I), and number of granulocytes (GRAs) (*p* < 0.01) (Figure 1J) in the peripheral blood. In healthy mice, PH alone enhanced the numbers of WBCs (*p* < 0.001) (Figure 1E), MONs (*p* < 0.001) (Figure 1F) and LYMs (*p* < 0.001) (Figure 1H).

**Figure 2:**
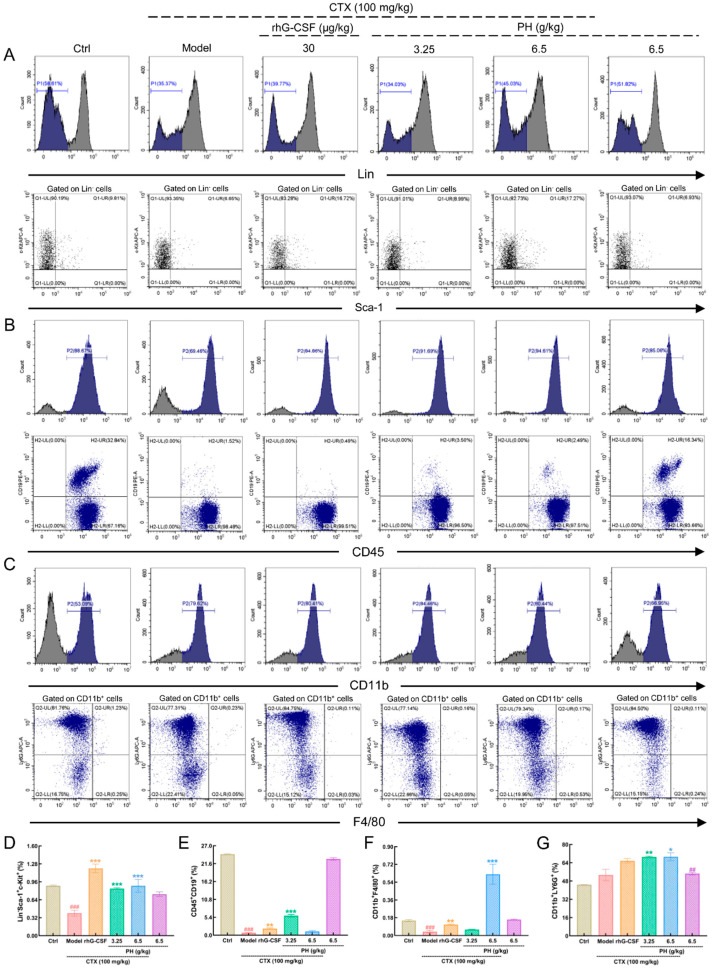
PH enhanced the levels of bone marrow mononuclear cells component. In bone marrow of the mice with hematopoietic dysfunction, PH enhanced (**A**) the levels of HSPC (represented by Lin^−^Sca-1^+^c-Kit^+^), (**B**) the levels of B lymphocytes (represented by CD45^+^CD19^+^), (**C**) the levels of macrophages (represented by CD11b^+^F4/80^+^), and granulocytes (represented by CD11b^+^Ly6G^+^), respectively. Quantitative analysis charts of (**D**) the levels of HSPCs, (**E**) the levels of B lymphocytes, (**F**) the levels of macrophages, and (**G**) granulocytes. Data are shown as the mean ± SD (*n* = 5 mice/group in triplicate) and determined via a one-way ANOVA test followed by a Tukey’s post hoc test comparison. ^##^ *p* < 0.01 and ^###^ *p* < 0.001 vs. control group, * *p* < 0.05, ** *p* < 0.01 and *** *p* < 0.001 vs. model group. CTX, cyclophosphamide; Ctrl, control; Model, hematopoietic dysfunction model; rhG-CSF, recombinant human granulocyte colony-stimulating factor; PH, Protein hydrolysate injection.

To assess changes in the cell composition of the bone marrow, the BMMNCs were isolated for flow cytometric analysis. Both PH and rhG-CSF significantly alleviated the reduction in Lin^−^ and Lin^−^/Sca-1^+^/c-Kit^+^ cells in the bone marrow of CTX-induced mice with hematopoietic dysfunction (Figure 2A,D). In the CTX-treated mice, the ratio of CD45^+^CD19^+^ cells was significantly lower than in healthy mice (*p* < 0.001) (Figure 2B,E). PH (3.25 g/kg) increased the proportion of CD45^+^CD19^+^ cells from 0.74% ± 0.11% to 5.88% ± 1.02% (*p* < 0.001). Meanwhile, treatment with rhG-CSF increased the proportion of CD45^+^CD19^+^ cells to 1.99% ± 0.5% (*p* < 0.01) (Figure 2B,E). CTX-treated mice had significantly reduced numbers of CD11b^+^F4/80^+^ cells (*p* < 0.001) (Figure 2C,F), while the proportion of CD11b^+^Ly6G^+^ cells remained unchanged (Figure 2C,G). PH increased the numbers of both CD11b^+^F4/80^+^ (*p* < 0.001) (Figure 2C,G) and CD11b^+^Ly6G^+^ (*p* < 0.01) cells (Figure 2C,G). However, rhG-CSF only increased the number of CD11b^+^F4/80^+^ cells (*p* < 0.01) (Figure 2C,F). In healthy mice treated with PH alone, only the proportion of CD11b^+^Ly6G^+^ cells was increased (*p* < 0.05) (Figure 2C,G).

### 3.3. PH Regulates Hematopoietic Cytokine Expression

**Figure 3:**
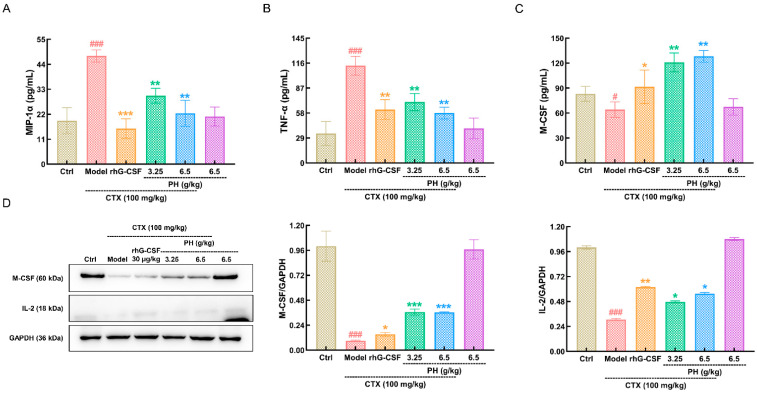
PH regulated the cytokines and proteins related to hematopoiesis. In CTX-induced mice with hematopoietic dysfunction, PH suppressed the levels of (**A**) MIP-1α and (**B**) TNF-α, and enhanced the levels of (**C**) M-CSF in serum (*n* = 6 mice/group in triplicate). (**D**) PH enhanced the expression levels of M-CSF and IL-2 in spleen (*n* = 6 mice/group in triplicate). Data are showed as the mean ± SD and analyzed via a one-way ANOVA test followed by a Tukey’s post hoc test comparison. Quantification data were normalized by GAPDH, and reported as the percentage of those from the corresponding control mice. ^#^ *p* < 0.05 and ^###^ *p* < 0.001 vs. control group, * *p* < 0.05, ** *p* < 0.01 and *** *p* < 0.001 vs. model group. CTX, cyclophosphamide; Ctrl, control; Model, hematopoietic dysfunction model; rhG-CSF, recombinant human granulocyte colony-stimulating factor; PH, Protein hydrolysate injection; M-CSF, macrophage colony stimulating factor; TNF-α, tumor necrosis factor-α; IL-2, interleukin-2.

To evaluate the protective effects of PH on hematopoietic function, we quantified the concentrations of various hematopoiesis-related cytokines in the serum and spleen. MIP-1α and TNF-α are negative regulators of hematopoiesis. The concentrations of MIP-1α (*p* < 0.001; Figure 3A) and TNF-α (*p* < 0.001; Figure 3B) were significantly increased, while the concentration of M-CSF (*p* < 0.05) was significantly decreased in CTX-treated mice compared with healthy mice (Figure 3C). These effects were suppressed following administration of PH (*p* < 0.01) or rhG-CSF (*p* < 0.05) (Figure 3A–C).

As positive regulators of hematopoiesis, M-CSF promotes bone resorption and IL-2 promotes B lymphocyte growth and differentiation. The expression levels of M-CSF and IL-2 in the spleen were analyzed by Western blotting. The levels of both were significantly lower (*p* < 0.001) in the spleens of CTX-treated mice than in healthy mice (Figure 3D). In contrast, both rhG-CSF and PH restored the levels of M-CSF (*p* < 0.05) and IL-2 (*p* < 0.05) (Figure 3D).

**Figure 4:**
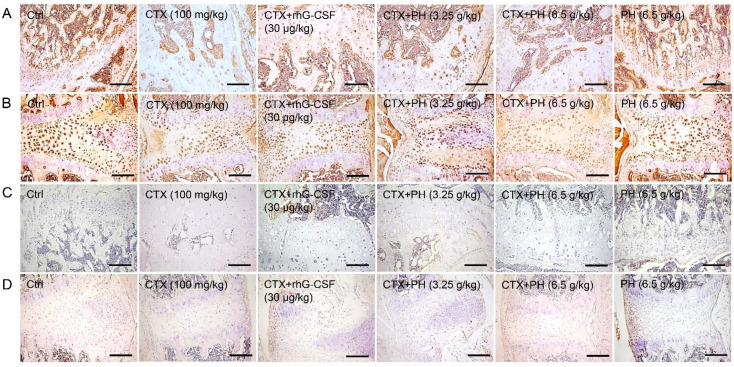
Histopathological observations of sternum and femoral condyles of mice with hematopoietic dysfunction. PH enhanced the expression levels of IL-2 in (**A**) femoral condyle and (**B**) sternum, but no positive staining of M-CSF was noted in (**C**) femoral condyle and (**D**) sternum detected by immunohistochemical staining under a light-microscope digital camera (100×, scale bar: 200 μm) (*n* = 3 mice/group in triplicate). CTX, cyclophosphamide; Ctrl, control; Model, hematopoietic dysfunction model; rhG-CSF, recombinant human granulocyte colony-stimulating factor; PH, Protein hydrolysate injection; M-CSF, macrophage colony stimulating factor; IL-2, interleukin-2.

To further investigate the roles of M-CSF and IL-2 in PH-induced improvements in hematopoietic function, their expression patterns in the femoral condyle and sternum were assessed by immunohistochemical staining. The expression levels of IL-2 in the femoral condyle and sternum of mice with hematopoietic dysfunction were lower than those in healthy mice, and these effects were suppressed following rhG-CSF and PH treatment (Figure 4A,B, Figure S4). However, no positive staining of M-CSF was noted in the femoral condyle or sternum among all groups (Figure 4C,D). PH alone was enhanced the expression of IL-2 in healthy mice (Figure 4A,B, Figure S4).

### 3.4. PH Regulates M-CSF to Improve Hematopoietic Function

**Figure 5:**
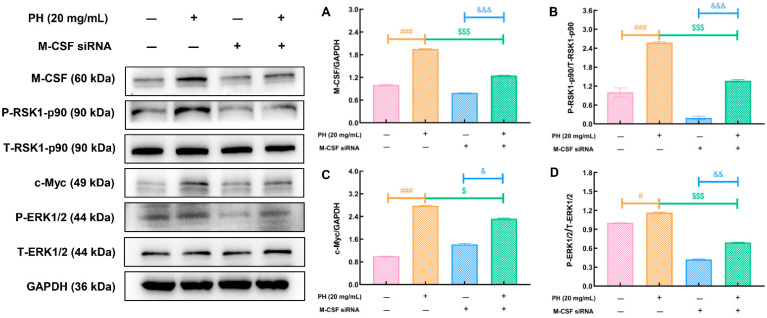
The regulation of PH on the expression of hematopoiesis-related proteins were partially abolished by M-CSF-siRNA transfection in K562 cells. The enhancement of PH on the expressions of (**A**) M-CSF, (**B**) P-RSK1-p90, (**C**) c-Myc, and (**D**) P-ERK1/2 in K562 cells were strongly abolished by M-CSF-siRNA transfection. Data are showed as the mean ± SD (*n* = 5 samples in triplicate) and analyzed via a one-way ANOVA test followed by a Tukey’s post hoc test comparison. Quantification data were normalized by GAPDH, and reported as the folds of those from the corresponding control cells. ^#^ *p* < 0.05 and ^###^ *p* < 0.001 vs. control group, ^$^ *p* < 0.05, ^$$$^ *p* < 0.001 vs. 20 mg/mL of PH treated K562 cells, ^&^ *p* < 0.05, ^&&^ *p* < 0.01, ^&&&^ *p* < 0.001 vs. M-CSF-siRNA treated K562 cells. CTX, cyclophosphamide; Ctrl, control; Model, hematopoietic dysfunction model; rhG-CSF, recombinant human granulocyte colony-stimulating factor; PH, Protein hydrolysate injection; M-CSF, macrophage colony stimulating factor; IL-2, interleukin-2; P-RSK1-p90, p90 ribosomal S6 kinases 1; P-ERK1/2, extracellular regulated protein kinases 1/2.

To confirm the relationship between M-CSF and PH, we used M-CSF siRNA to suppress the expression of the M-CSF gene in K562 cells. In PH-treated K562 cells, the expression of M-CSF (*p* < 0.001) (Figure 5A), P-RSK1-p90 (*p* < 0.001) (Figure 5B), c-Myc (*p* < 0.001) (Figure 5C), and P-ERK (*p* < 0.001) (Figure 5D) was significantly upregulated. However, these effects were significantly alleviated following transfection of M-CSF siRNA (*p* < 0.05) (Figure 5). Compared with K562 cells only treated with M-CSF siRNA, the expression of M-CSF (*p* < 0.001), P-RSK1-p90 (*p* < 0.001), c-Myc (*p* < 0.05), and P-ERK (*p* < 0.01) had also been increased by PH (Figure 5).

## 4. Discussion

As a nutritional drug in China, PH is used to treat severe amino acid deficiency and hypoproteinemia caused by various diseases and to promote tissue healing and restore normal physiological function. In this study, we identified a protective effect of PH on hematopoietic function in a mouse model of CTX-induced hematopoietic dysfunction and in K562 cells. In mice with hematopoietic dysfunction, PH upregulated the expression of IL-2 and M-CSF and altered the composition of the bone marrow hematopoietic cell population. The peptides and amino acids that compose PH are hematopoietic substances that can enhance body metabolism and promote the production, differentiation, maturation, and release of blood cells. Based on the standards of Chinese Pharmacopoeia, the total nitrogen content of PH should be 0.6–0.8% (g/mL) and the α-amino acid content should be higher than 60%. However, no systemic analysis of the composition of PH has been carried out previously. We report that PH contains 17 different amino acids, some of which are associated with regeneration of hematopoietic cells and the synthesis of anti-inflammatory mediators [28]. Leucine is thought to improve hematopoietic function [29]. Valine is thought to play a role in maintaining the number of HSCs. Depletion of valine has been reported to reduce the total number of HSCs [30]. Fe-Gly, or Gly chelated with iron, is thought to increase the percentage of Th1 cells and enhance the production of cytotoxic CD8^+^ T cells and IL-2 [31]. The composition of PH likely is related directly to its beneficial effects on hematopoietic function.

The spleen and thymus are central hematopoiesis-related organs. Extramedullary hematopoiesis (EMH) refers to the formation and development of blood cells outside the medullary space of the bone marrow [32]. Patients with bone marrow diseases often experience EMH [33]. As a common site of EMH, the spleen can provide a site for hematopoiesis [34]. In contrast, T lymphocytes are generated in the thymus [35]; however, no self-renewing progenitor cells reside in the thymus, and they must migrate from the bone marrow [36]. Thus, the BM microenvironment plays an important role in regulating hematopoietic function [37]. When hematopoietic dysfunction occurs, especially chemotherapy-induced myelosuppression, an increase in adipose and spleen tissue and a decrease in hematopoietic tissue and megakaryocytes is seen [38], and nucleated myeloid cells are replaced by vacuolation in the bone marrow [39]. According to previous research and our data, these pathologic changes can be normalized by rhG-CSF treatment, a commonly used agent in myelosuppression treatment [40]. Similarly, in mice with CTX-induced hematopoietic dysfunction, PH alleviated these pathologic changes in the spleen, thymus and even the bone marrow, suggesting a significant ability to improve hematopoietic function.

In mice with CTX-induced hematopoietic dysfunction, PH alleviated the pathologic changes seen in the physiological indexes of peripheral blood. As WBCs are the main component of the human immune system and peripheral blood, a reduced WBCs count is one of the main manifestations of hematopoietic dysfunction [41]. Recovery of hematopoiesis can be confirmed by increasing levels of WBCs in the peripheral blood [42]. The number of WBCs in the PH-treated mice increased significantly, suggesting improved immune function. Interestingly, in healthy mice, PH increased the numbers of monocytes and lymphocytes, but failed to influence their percentages in peripheral blood, suggesting that PH increased the total number of blood cells uniformly.

HSPCs are located in the bone marrow, and the cells that they produce enter the peripheral circulatory system [43]. Chemotherapy-induced myelosuppression can reduce the self-renewal and differentiation capacity of HSPCs and thus the total number of BMMNCs [44]. PH treatment increased the numbers of HSPCs, B lymphocytes, macrophages, and granulocytes in the bone marrow of mice with hematopoietic dysfunction. We also noted that PH alone increased the number of granulocytes in healthy mice. An increased number of granulocytes may cause various allergic diseases in patients. This phenomenon may help us to reveal the adverse reactions caused by PH applied in clinics, such as anaphylaxis, which has been reported to be related to anomalous level of granulocytes [45]. These issues need further investigation not only in animal experiments, but also in clinical trials.

In the serum of mice with hematopoietic dysfunction, PH ameliorated increases in the levels of TNF-α and MIP-1α. TNF-α is a negative regulator of hematopoiesis and can inhibit the proliferation of HPCs [46] and reduce the proliferation and differentiation of HSCs [47]. MIP-1α, derived from monocytes, neutrophils, and lymphocytes, can inhibit HSPCs proliferation [48]. Accordingly, PH was found to promote the proliferation of HSPCs by decreasing the levels of TNF-α and MIP-1α.

By using several experimental methods, we confirmed that PH increased the expression of IL-2 and M-CSF in mice with hematopoietic dysfunction. In the healthy mice, PH alone also enhanced the expressions of IL-2 in femoral condyle and sternum. As one of the key regulators of hematopoietic cell proliferation, IL-2 stimulates the phosphorylation of ERK [49,50]. M-CSF activates MAPK-related pathways in bone marrow progenitor cells [51]. As a member of the MAPK family, phosphorylation-activated ERK1/2 mediates the activation of the transcription factor c-Myc and the expression of cytokines involved in cell proliferation [52]. c-Myc plays a role in hematopoietic homeostasis, and its upregulation is essential for initiating the differentiation of HSPCs [53]. Expression of c-Myc can be upregulated by IL-2 [54]. Both c-Myc and RSK1-p90 are downstream targets of ERK1/2 [55,56]. Furthermore, the upregulated expression levels of M-CSF, P-ERK1/2, P-RSK1-p90, and c-Myc seen in K562 cells exposed to PH were reversed following transfection with M-CSF siRNA. According to a previous study, phosphorylated ERK1/2 can enhance the expression of IL-2 [57]. Therefore, M-CSF may positively regulate the production of IL-2 via ERK, further confirming the central role of M-CSF in PH-mediated improvements in hematopoietic function.

There are some limitations in this study. For example, the active molecules in PH, a multi-peptide drug, are still unclear. It is hard for us to confirm which components, polypeptides or amino acids, play the important role in regulating M-CSF. Additionally, although we found that the effects of PH on hematopoietic function are at least partially related to the regulation of M-CSF levels, our data also suggest the potential involvement of M-CSF-independent pathways. Future studies should identify the specific signaling pathways involved. Finally, PH may influence several different cell types related to hematopoietic function; however, we failed to confirm the specific cell types that are directly affected by PH.

## 5. Conclusions

Based on the systematic analysis on the components of PH, its improvement on hematopoietic function was confirmed in myelosuppressed mice, evidenced by the alleviation of pathological changes on BM, the restoration of peripheral blood cell concentrations, and the increment on the numbers of HSPCs, B lymphocytes, macrophages, and granulocytes in the BM. Further data suggest that PH improved the hematopoietic function of CTX-treated mice via the regulation of M-CSF. Our data provide an experimental basis for the clinical application of PH to improve hematopoietic function, especially for chemotherapeutic cancer patients.

## Figures and Tables

**Table 1:** Antibody information.

Description	Item Number	Molecular Weight (kDa)	Dilution Rate	Applications
Macrophage colony-stimulating factor (M-CSF) ^a^	ab233387	60	1:1000/1:500 (WB/IHC)	WB, IHC
p90 ribosomal S6 kinases 1 (RSK1-p90) ^a^	ab32526	90	1:1000	WB
P-RSK1-p90 (phospho T359/S363) ^a^	ab32413	90	1:1000	WB
c-Myc ^b^	A1309	49	1:1000	WB
Extracellular regulated protein kinases (ERK)1/2 ^a^	ab36991	44	1:2000	WB
P-ERK1/2 (phospho T202/T185) ^b^	AP0485	44	1:1000	WB
Interleukin (IL)-2 ^b^	A16317	18	1:1000/1:200 (WB/IHC)	WB, IHC
Glyceraldehyde-3-phosphate dehydrogenase (GAPDH) ^c^	E-AB-20032	36	1:1000	WB
Goat Anti Mouse IgG (H+L) (peroxidase/HRP conjugated) ^c^	E-AB-1001	-	1:1000	WB
Goat Anti Rabbit IgG (H+L) ^c^	E-AB-1025	-	1:1000	WB
FITC anti-mouse Lineage Cocktail with Isotype Ctrl ^d^	78022	-	20 µL/10^6^ cells in 100 µL	FC
PE anti-mouse Ly-6A/E (Sca-1) Clone: D7 ^d^	108108	-	2.5 mL/10^6^ cells in 100 µL	FC
APC anti-mouse CD117 (c-Kit) Clone:2B8 ^d^	105812	-	5 μL/10^6^ cells in 100 µL	FC
PE anti-mouse F4/80 Clone:BM8 ^d^	123110	-	5 μL/10^6^ cells in 100 µL	FC
FITC anti-mouse/human CD11b Clone:M1/70 ^d^	101205	-	0.5 μL/10^6^ cells in 100 µL	FC
APC anti-mouse Ly-6G Clone:1A8 ^d^	127613	-	0.3 μL/10^6^ cells in 100 µL	FC
FITC-CD3e Monoclonal Antibody Clone:145-2C11 ^e^	11-0031-82	-	1 μL/10^6^ cells in 100 µL	FC
PE anti-mouse CD19 Clone:6D5 ^d^	115507	-	1.25 μL/10^6^ cells in 100 µL	FC
APC anti-mouse CD45 Clone:30-F11 ^d^	103112	-	1.25 μL/10^6^ cells in 100 µL	FC
FITC-conjugated anti-rat IgG2a ^d^	400505	-	0.3 μL/10^6^ cells in 100 µL	FC
PerCP-conjugated anti-rat IgG2b ^d^	400629	-	1.25 μL/10^6^ cells in 100 µL	FC
APC-conjugated anti-rat IgG2b ^d^	400611	-	5 μL/10^6^ cells in 100 µL	FC
FITC-conjugated anti-rat IgG2b ^d^	400605	-	0.5 μL/10^6^ cells in 100 µL	FC

Antibodies were purchased from ^a^ Abcam China (Shanghai, China), ^b^ ABclonal Technology Co., Ltd. (Wuhan, China), ^c^ Elabscience Biotechnology Co., Ltd. (Wuhan, China), ^d^ Biolegend (San Diego, CA, USA), ^e^ Invitrogen, Thermo Fisher Scientific (Carlsbad, CA, USA). WB, Western blot; IHC, Immunohistochemistry; FC, Flow cytometry.

**Table 2:** The molecular mass distribution of PH.

Distribution of Molecular Mass (g/mol)	Percentage (%)
185.0–264.0	38.6
264.0–475.0	58.9
475.0–622.0	2.5

PH, Protein hydrolysate injection.

**Table 3:** The amino acid contents of PH.

Amino Acid	Contents (g/100 g)
Aspartic Acid	0.39
Threonine	0.18
Serine	0.19
Glutamic acid	**0.64**
Glycine	0.10
Alanine	0.27
Cystine	0.019
Valine	0.30
Methionine	0.074
Isoleucine	0.22
Leucine	**0.42**
Tyrosine	0.034
Phenylalanine	0.075
Lysine	**0.45**
Histidine	0.10
Arginine	0.068
Proline	**0.41**

Bold means that the content of amino acid is higher than 0.4 g/100 g. PH, Protein hydrolysate injection.

## Data Availability

The data that support the findings of this study are available from the corresponding author upon reasonable request. The source data underlying Table S1 and Figures S1–S4 are provided as a Source Data file.

## Supplementary Materials

The following are available online at www.mdpi.com/article/10.3390/cells10102776/s1, Figure S1: The animal experimental schemes flow charts, Figure S2: Define peaks of PH, Figure S3: H&E staining was used to evaluate pathological alterations, Figure S4, Quantitative graph of the expression of IL-2 in the femoral condyle and sternum of mice with hematopoietic dysfunction, Table S1: The effects of PH and rhG-CSF on body weight and organ indexes of CTX-induced hematopoietic dysfunction mice.

## Abbreviations

ATCC, American Type Culture Collection; BCA, bicinchoninic acid; BM, bone marrow; BMMNCs, bone marrow-derived mononuclear cells; CTX, cyclophosphamide; ELISA, enzyme-linked immunosorbent assay; EMH, extramedullary hematopoiesis; EPO, erythropoietin; ERK1/2, extracellular regulated protein kinases 1/2; FBS, fetal bovine serum; FC, flow cytometry; GAPDH, glyceraldehyde-3-phosphate dehydrogenase; GPI, glycosylphosphatidylinositol; GRAs, granulocytes; HPLC, high-performance liquid chromatograph; HPCs, hematopoietic progenitor cells; HSCs, hematopoietic stem cells; HSPCs, hematopoietic stem cells and progenitor cells; HRP, horseradish peroxidase; H&E, hematoxylin and eosin; IHC, immunohistochemistry; IL-2, interleukin-2; LYM, lymphocytes; M-CSF, macrophage colony stimulating factor; MIP-1α, macrophage inflammatory protein-1α; MON, monocytes; NS, normal saline; PH, protein hydrolysate injection; PBS, phosphate buffered saline; PVDF, polyvinylidene difluoride; rhG-CSF, recombinant human granulocyte colony-stimulating factor; RPMI, roswell park memorial institute; SD, standard deviation; RSK1-p90, p90 ribosomal S6 kinases 1; SPF, specific pathogen-free; TBS, triethanolamine-buffered saline; TBST, triethanolamine-buffered saline containing Tween 20; TNF-α, tumor necrosis factor-α; WB, Western blot; WBC, white blood cell.
