# Supplementary material for: The Involvement of Macrophage Colony Stimulating Factor on Protein Hydrolysate Injection Mediated Hematopoietic Function Improvement
Source: Cells. 2021 Oct 16;10(10):2776. doi: 10.3390/cells10102776 (PMC8534652; doi:10.3390/cells10102776)
Supplement: Supplementary file 1 [file cells-10-02776-s001.zip › cells-1368947-supplementary.pdf]

cells

MDPI

Article

The Involvement of Macrophage Colony Stimulating Factor on Protein Hydrolysate Injection Mediated Hematopoietic Function Improvement

Shimiao Wang [1,2#], Yuchong Zhang [2#], Weiqi Meng [1], Yihao Dong [2], Sujie Zhang [2], Lesheng Teng [1], Yang Liu [2], Lanzhou Li [1,2,*] and Di Wang [1,2,*]

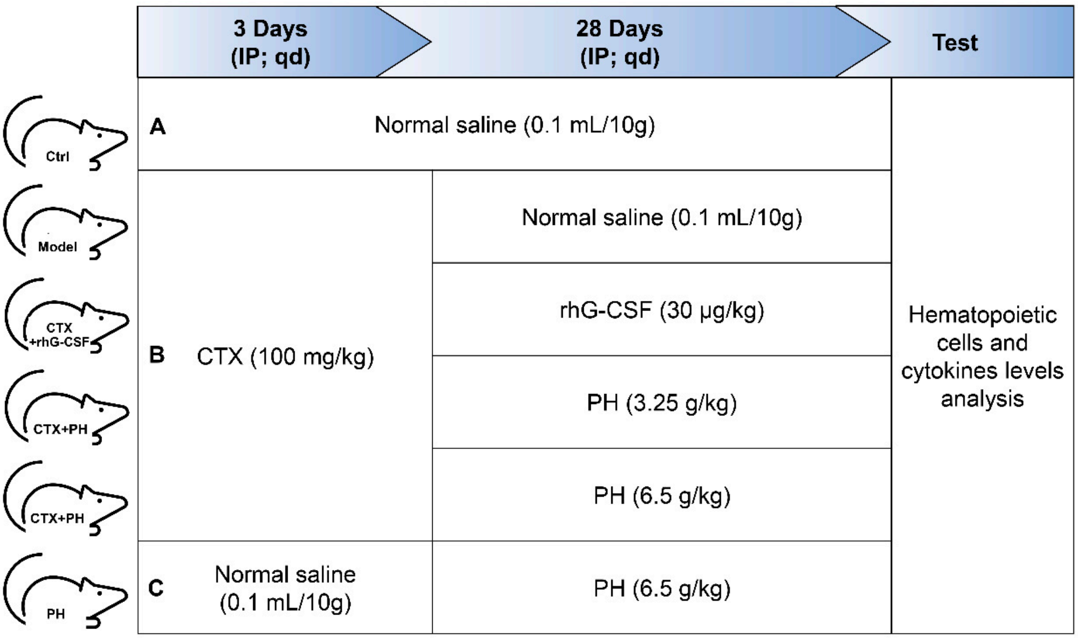

Supplementary Figure S1 The animal experimental schemes flow charts. CTX, cyclophosphamide; Ctrl, control; Model, hematopoietic dysfunction model; NS, normal saline; rhG-CSF, recombinant human granulocyte colony-stimulating factor; PH, protein hydrolysate injection.

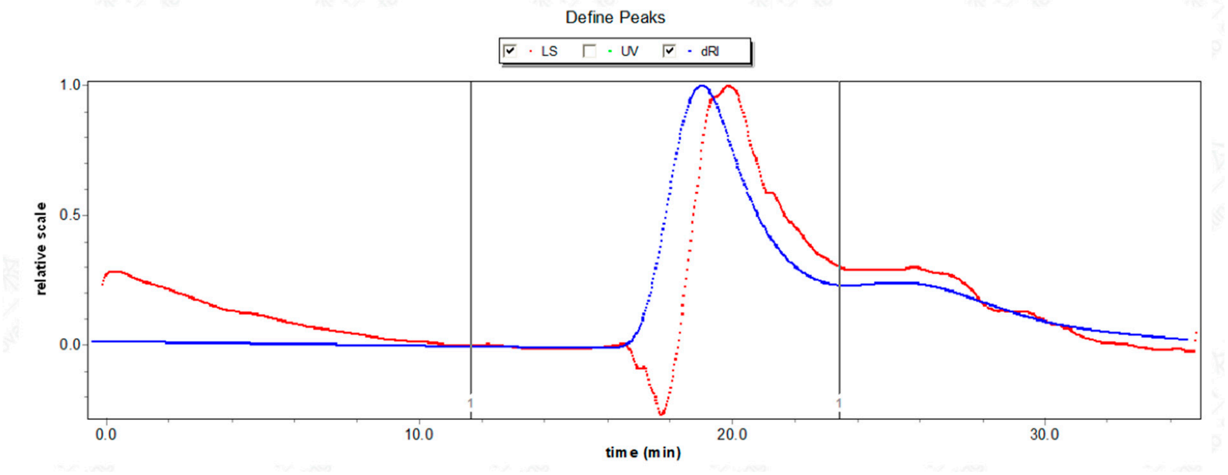

Supplementary Figure S2 Define peaks of PH.

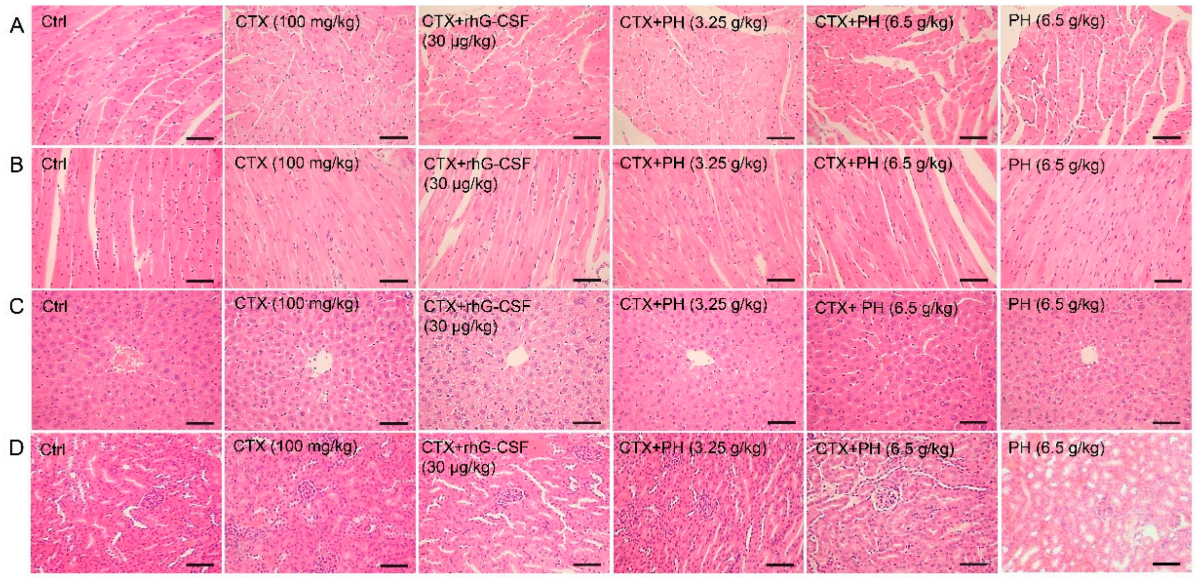

Supplementary Figure S3 H&E staining was used to evaluate pathological alterations of (A) heart (cross section), (B) heart (longitudinal section), (C) liver and (D) kidney under a light-microscope digital camera (200 ×; scale bar, 100 μm) (n = 3 mice/group in triplicate). CTX, cyclophosphamide; Ctrl, control; Model, hematopoietic dysfunction model; rhG-CSF, recombinant human granulocyte colony-stimulating factor; PH, Protein hydrolysate injection.

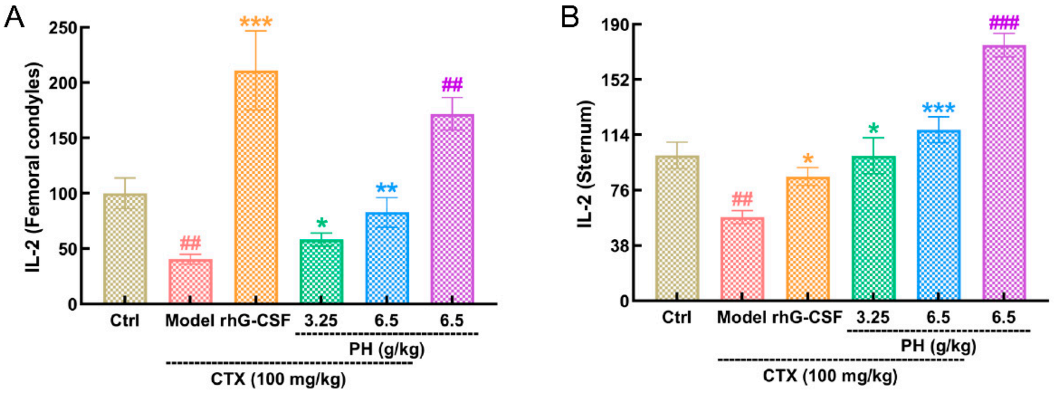

Supplementary Figure S4 Quantitative graph of the expression of IL-2 in the femoral condyle and sternum of mice with hematopoietic dysfunction. Histopathological observations of sternum and femoral condyles of mice with hematopoietic dysfunction. PH enhanced the expression levels of IL-2 in (A) femoral condyle and (B) sternum detected by immunohistochemical staining. Data are showed as the mean ± SD (n = 3 mice/group in triplicate) and determined via a one-way ANOVA test followed by a Tukey's post hoc test comparison. $^{\#\#}p < 0.01$ and $^{\#\#\#}p < 0.001$ vs. control group, $^{*}p < 0.05$, $^{**}p < 0.01$ and $^{***}p < 0.001$ vs. model group. CTX, cyclophosphamide; Ctrl, control; Model, hematopoietic dysfunction model; rhG-CSF, recombinant human granulocyte colony-stimulating factor; PH, Protein hydrolysate injection; IL-2, interleukin 2.

Supplementary Table 1 The effects of PH and rhG-CSF on body weight and organ indexes of CTX induced hematopoietic dysfunction mice.

Data are showed as the means ± S.D. (n=15 mice/group). CTX, cyclophosphamide; Ctrl, control; Model, hematopoietic dysfunction model; rhG-CSF, recombinant human granulocyte colony-stimulating factor; PH, Protein hydrolysate injection. $^{\#\#}p < 0.01$ and $^{\#\#\#}p < 0.001$ vs. Ctrl group, $^{*}p < 0.05$, $^{**}p < 0.01$ and $^{***}p < 0.001$ vs. Model group. CTX,

		Ctrl	CTX (100 mg/kg)				PH (6.5 g/kg)
			Model	rhG-CSF (30 μg/kg)	PH (3.25 g/kg)	PH (6.5 g/kg)	
Body weight (g)	1st day	22.9±2.1	23.5±2.1	23.5±1.0	23.5±1.4	23.7±0.9	23.3±0.9
	4th day	24.3±2.4	22.2±1.5$^{\#\#}$	23±1.3	22.3±1.4	22.5±1.1	24.6±0.9
	11th day	25.7±1.7	22.7±1.6$^{\#\#\#}$	21.5±2.0	23.3±1.6	23.4±1.2	26.2±1.0
	18th day	26.0±2.3	23.4±1.6$^{\#\#}$	24.5±1.0	24.4±1.4	24.5±1.2	27.4±1.0
	25th day	26.8±2.7	21.6±1.7$^{\#\#\#}$	23.7±1.4**	22.7±1.3	23.3±1.6*	27.9±1.2
	32nd day	27.4±1.9	20.7±2.7$^{\#\#\#}$	23.1±1.4**	23.6±1.5**	23.0±1.4**	28.5±1.4
Organ index (%)	Liver index	48.9±3.2	49.7±3.0	52.0±3.5	51.7±2.9	51.9±3.9	48.6±3.8
	Kidney index	14.5±0.9	13.6±1.2	13.5±0.7	12.0±1.6	13.9±1.6	14.0±0.8
	Spleen index	3.5±0.4	13.4±0.8$^{\#\#\#}$	12.2±1.2	9.8±1.5***	10.1±1.0***	3.3±0.2
	Thymus index	1.6±0.2	0.5±0.3$^{\#\#\#}$	1.0±0.5**	0.8±0.4*	0.6±0.3	1.4±0.2

cyclophosphamide; Ctrl, control; Model, hematopoietic dysfunction model; rhG-CSF, recombinant human granulocyte colony-stimulating factor; PH, Protein hydrolysate injection.
